# Non-classic radiation-induced liver disease after intensity-modulated radiotherapy for Child–Pugh grade B patients with locally advanced hepatocellular carcinoma

**DOI:** 10.1186/s13014-023-02232-5

**Published:** 2023-03-08

**Authors:** Jian-Xu Li, Rui-Jun Zhang, Mo-Qin Qiu, Liu-Ying Yan, Mei-Ling He, Mei-Ying Long, Jian-Hong Zhong, Hai-Yan Lu, Hong-Mei Zhou, Bang-De Xiang, Shi-Xiong Liang

**Affiliations:** 1 Department of Radiation Oncology, Guangxi Medical University Cancer Hospital, Nanning, 530021 China; 2 Department of Respiratory Oncology, Guangxi Medical University Cancer Hospital, Nanning, China; 3 Department of General Affairs, Guangxi Medical University Cancer Hospital, Nanning, China; 4 School of Public Health, Guangxi Medical University, Nanning, 530021 China; 5 Department of Hepatobiliary Surgery, Guangxi Medical University Cancer Hospital, Nanning, 530021 China

**Keywords:** Child–Pugh grade B, Hepatocellular carcinoma, Intensity-modulated radiotherapy, Nomogram, Radiation-induced liver disease

## Abstract

**Background:**

The incidence of classic radiation-induced liver disease (cRILD) has been significantly reduced. However, non-classic radiation-induced liver disease (ncRILD) remains a major concern following radiotherapy in patients with hepatocellular carcinoma (HCC). This study evaluated the incidence of ncRILD following intensity-modulated radiotherapy (IMRT) for Child–Pugh grade B (CP-B) patients with locally advanced HCC and established a nomogram for predicting ncRILD probability.

**Methods:**

Seventy-five CP-B patients with locally advanced HCC treated with IMRT between September 2014 and July 2021 were included. The max tumor size was 8.39 cm ± 5.06, and the median prescribed dose was 53.24 Gy ± 7.26. Treatment-related hepatotoxicity was evaluated within three months of completing IMRT. A nomogram model was formulated to predict the probability of ncRILD, using univariate and multivariate analysis.

**Results:**

Among CP-B patients with locally advanced HCC, ncRILD occurred in 17 (22.7%) patients. Two patients (2.7%) exhibited a transaminase elevation of ≥ G3, fourteen (18.7%) exhibited a Child–Pugh score increase of ≥ 2, and one (1.3%) demonstrated both a transaminase elevation of ≥ G3 and a Child–Pugh score increase of ≥ 2. No cRILD cases were observed. A mean dose to the normal liver of ≥ 15.1 Gy was used as the cutoff for ncRILD. Multivariate analysis revealed that the prothrombin time before IMRT, tumour number, and mean dose to the normal liver were independent risk factors for ncRILD. The nomogram established on the basis of these risk factors displayed exceptional predictive performance (AUC = 0.800, 95% CI 0.674–0.926).

**Conclusions:**

The incidence of ncRILD following IMRT for CP-B patients with locally advanced HCC was acceptable. A nomogram based on prothrombin time before IMRT, tumour number, and mean dose to the normal liver accurately predicted the probability of ncRILD in these patients.

**Supplementary Information:**

The online version contains supplementary material available at 10.1186/s13014-023-02232-5.

## Background

Hepatocellular carcinoma (HCC) accounts for about 75–85% of primary liver cancer cases with the third-highest fatality rate among malignant diseases worldwide [1]. HCC mostly occurs in patients with liver cirrhosis [2]. Child–Pugh grade is the most widely used to rank liver function. Patients with Child–Pugh grade B (CP-B) liver cirrhosis have limited therapeutic options because of the risk of impaired liver function [3]. Treatments for the HCC patients with CP-B liver cirrhosis remains a controversial topic [4].

Radiotherapy (RT), including intensity-modulated radiotherapy (IMRT) and stereotactic body radiation therapy (SBRT), is commonly used to treat HCC [5, 6], and the American Association for the Study of Liver Diseases recommend RT as a standard treatment for HCC [7]. Radiation-induced liver disease (RILD) is the major form of dose-limiting toxicity associated with RT treatment in HCC [8]. RILD is divided into classic RILD (cRILD) and non-classic RILD (ncRILD) [9]. cRILD has been well described, and the incidence of cRILD has been significantly decreased by technological advances in RT, especially IMRT. However, there is still risk of developing radiation-induced hepatotoxicity for the patients, therefore many researches have described the minor hepatotoxicity as ncRILD [10]. Patients with HCC and pre-existing liver disease, such as CP-B liver function, are more susceptible to hepatic radiation toxicity [11]. Conversely, the probability of hepatic radiation toxicity is difficult to predict for the CP-B patients with locally advanced HCC because of the small number [4, 12].

Recently, acceptable hepatotoxicity levels have been reported following SBRT in patients with CP-B HCC [13, 14]. However, it is unclear whether the benefit of IMRT outweigh RILD in CP-B patients with locally advanced HCC. Therefore, our study aimed to evaluate the incidence of RILD after IMRT in CP-B patients with locally advanced HCC and to establish a nomogram for predicting the probability of RILD.

## Methods

### Study design and patients

The retrospective study was performed based on the ethical guidelines of the declaration of Helsinki, and approved by the ethics committee of Guangxi Medical University Cancer Hospital (LW2022053). From September 2014 to July 2021, 96 CP-B patients with locally advanced HCC were administered IMRT. A total of 75 patients met the following chief eligibility criteria for inclusion in the study: (1) HCC diagnosed according to histopathology or clinical criteria on the basis of imaging-based characteristics [15]; (2) CP-B liver function; (3) Eastern Cooperative Oncology Group performance status score of 0–2; (4) unresectable, locally advanced disease; (5) prior treatment with IMRT and available dosimetry data; (6) tumour not suitable for radical cures such as hepatic resection or local ablation; (7) an follow-up time of ≥ 3 months if possible; and (8) complete clinical information and follow-up information. Major exclusion criteria were patients unfinished IMRT, lost to laboratory testing, combined intrahepatic cholangiocarcinoma, and lost to dosimetric data.

### Radiotherapy technique

The contrast-enhanced computed tomography (CT) scans for the dose calculation plans were underwent by positioning the patients in supine with their arms overhead during free quiet breathing. Gross tumour volume (GTV) including macrovascular invasion (MVI) was clearly delineated using the contrast-enhanced CT and CT–magnetic resonance imaging fusion. The GTV was expanded to 4–5 mm to establish the clinical target volume (CTV). The planning target volume included the CTV plus a 5–10 mm margin, implemented to account for respiratory motion and setup uncertainty. All target areas and organs at risk were contoured using the Pinnacle 3 system (Philips, Netherlands) or MIM 6.8 system (MIM, USA). IMRT plans were designed using the Monaco treatment planning system (version 5.1) or the Pinnacle 3 system (Philips, Netherlands). Among the 75 patients, the median total GTV dose was 53.24 Gy ± 7.26 Gy (mean ± standard deviation), with a median of 2.96 Gy ± 0.84 Gy per fraction; each fraction was 3 to 5 days a week. IMRT was administered to all patients using a linear accelerator with 6 MV X-rays (ELEKTA Versa-HD or ELEKTA Synergy, Sweden).

### Follow-up and RILD assessment

After IMRT, all patients were followed up at 1 month, every 3 months for 2 years, every 6 months for 5 years, and every 1 year thereafter. The patients underwent CT and/or MRI within 1 month before the initiation of IMRT and every 2–3 months after IMRT. cRILD involves a serum alkaline phosphatase (ALP) level of more than twice the baseline value or upper limit of the normal, with anicteric hepatomegaly and ascites [16]. Various definitions for ncRILD have been proposed; a liver transaminase elevation of at least five times the upper limit of the normal or baseline value, or an increase of 2 or more in the Child–Pugh score within 3 months of RT completion, is a widely used index applied [10]. Laboratory tests, including tests for aspartate aminotransferase (AST), alanine aminotransferase (ALT), ALP, albumin (ALB), total bilirubin (TB), prothrombin time (PT), alpha fetoprotein (AFP), white blood cells (WBC), hemoglobin (HGB), platelets (PLT), absolute neutrophil count (ANC), absolute lymphocyte count (ALC) and hepatitis B virus (HBV) DNA, were conducted 1 week before IMRT (Table 1). Laboratory tests for AST, ALT, ALP, ALB, TB, and PT were conducted monthly after IMRT. Common Terminology Criteria for Adverse Events version 5.0 (CTCAE 5.0) was used to evaluate hepatotoxicity. Liver function was evaluated by calculating albumin-bilirubin (ALBI) score and Child–Pugh grade. Patients with hepatotoxicity attributed to disease progression and/or HBV replication were excluded. Disease progression was defined as the progression of liver target lesions using CT or MR images according to the Response Evaluation Criteria in Solid Tumours version 1.1. HBV reactivation was defined as a tenfold or greater increase in HBV DNA levels from the baseline [17].

**Table 1:** Baseline characteristics of patients

Characteristic	Value
*Sex*	
Male	69 (92.0)
Female	6 (8.0)
Age, median (range, years)	50 (29–72)
Bodyweight (range, kg)	60 (35–91)
Radiographic liver cirrhosis	38 (50.7)
*Hepatitis etiology*	
Hepatitis B virus	66 (88.0)
Hepatitis C virus	3 (4.0)
Other	7 (9.3)
*ECOG PS*	
0	18 (24.0)
1	56 (74.7)
2	1 (1.3)
*Child–Pugh score*	
7	59 (78.7)
8	13 (17.3)
9	3 (4.0)
ALBI score	-1.740 ± 0.36
*ALBI grade*	
2	59 (78.7)
3	16 (21.3)
*Alpha fetoprotein, ≥ 400 ng/ml*	
Yes	37 (49.3)
No	38 (50.7)
Total bilirubin (μmol/L)	24.81 ± 21.36
Albumin (g/L)	30.22 ± 3.37
PT (sec)	13.35 ± 1.65
AST (U/L)	89.37 ± 120.78
ALT (U/L)	60.13 ± 69.90
ALP (U/L)	176.64 ± 105.56
Max tumor size (cm)	8.39 ± 5.06
*Tumor number*	
< 3	36 (48.0)
≥ 3	39 (52.0)
*Macrovascular invasion*	
Yes	53 (70.7)
No	22 (29.3)
*Extrahepatic metastasis*	
Yes	35 (46.7)
No	40 (53.3)
*BCLC stage*	
A	6 (8.0)
B	2 (2.7)
C	67 (89.3)
Total dose (Gy)	53.33 ± 7.08
Dose per fraction (Gy)	2.96 ± 0.84
GTV (cc/ml)	749.23 ± 729.88
EQ D2^8^ (Gy)	59.23 ± 10.42
NLV (cc/ml)	911.56 ± 269.88
Dmean (Gy)	17.54 ± 5.26
*Prior treatment*	
TACE	56 (74.7)
RFA	6 (8.0)
Surgical resection	26 (34.7)
Systemic therapy	18 (24.0)

Data are mean ± standard deviation, median (IQR) or N (%), unless indicated

ALBI, albumin–bilirubin; ALC, absolute lymphocyte count; ALP, alkaline phosphatase; ALT, alanine aminotransferase; ANC, absolute neutrophil count; AST, aspartate aminotransferase; BCLC, Barcelona Clinic Liver Cancer; Dmean, mean dose to the normal liver; ECOG PS, Eastern Cooperative Oncology Group-performance status; EQD2, equivalent dose in 2‑Gy fractions; GTV, gross tumor volume; HGB, hemoglobin; NLV, normal liver volume; PLT, platelets; PT, prothrombin time; RFA, radiofrequency ablation; RT, radiotherapy; TACE, transcatheter chemoembolization; WBC, white blood cells; ^8^, using LQ model, α/β = 8 Gy

### Statistical analysis

The overall survival (OS) was defined as the date of the informed consent for radiotherapy to death from any cause. Survival curves for patients with HCC with and without RILD were constructed using the Kaplan–Meier method. The cut-off values for the risk factor parameters in the RILD model were determined using Youden’s index [18] and receiver operating characteristic (ROC) curve analysis. The significant variables identified by logistic regression analysis were entered into univariate and multivariate logistic regression analysis to identify patient characteristics associated with RILD. The univariate included the following variables: Sex, age, bodyweight, radiographic liver cirrhosis, hepatitis etiology, ECOG PS, Child–Pugh Score, ALBI score, ALBI Grade, TB, ALB, PT, AST, ALT, ALP, WBC, HGB, PLT, ANC, ALC, AFP, max tumor size, tumor number, MVI, extrahepatic metastasis, BCLC stage, dose per fraction, GTV, equivalent dose in 2 Gy per fraction (EQD2), normal liver volume (NLV), mean dose to the normal liver (D_mean_), transcatheter chemoembolization, radiofrequency ablation, and surgical resection, systemic therapy. The multivariate included the following variables: including PT, tumor number, and Dmean.

A nomogram for predicting RILD probability was formulated using the results of the multivariate logistic regression analysis. The area under the ROC curve (AUC) and the calibration curve were used to evaluate nomogram performance. In addition, nomogram calibration was validated by conducting 1000 bootstrap resamples. Statistical significance was set at *p* < 0.05. R version 4.0.5 (http://www.r-project.org/) was used for all statistical analyses.

## Results

### Patients

**Fig. 1:**
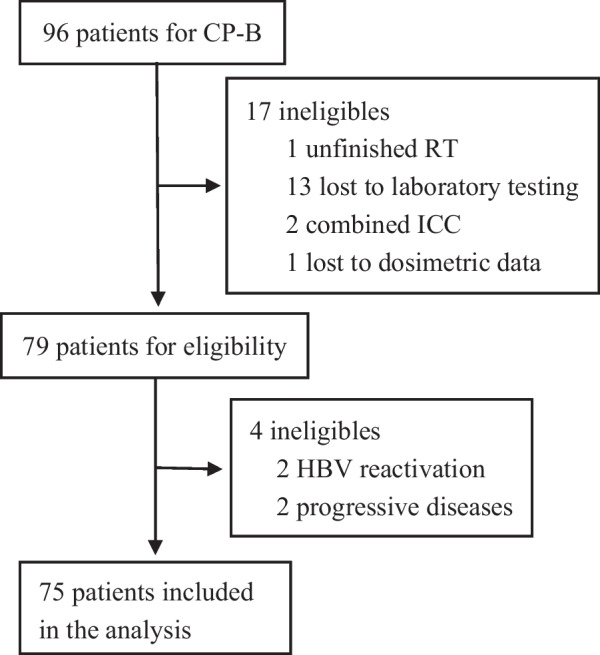
Flow diagram of the study. CP-B, Child–Pugh grade B; HBV, hepatitis B virus; ICC, intrahepatic cholangiocarcinoma; RILD, radiation-induced liver disease; RT, radiotherapy

From September 2014 to July 2021, a total of 75 CP-B patients with locally advanced HCC with a base-line CP score of B7 (59 patients), B8 (13 patients) and B9 (3 patients) met the criteria and were enrolled (Fig. 1). The baseline characteristics of the patients are described in Table 1. Briefly, 88.0% patients had chronic hepatitis B virus (HBV) etiology and 50.7% presented with radiographic liver cirrhosis; the max tumor size was 8.39 cm ± 5.06; 89.3% patients were diagnosed with Barcelona Clinic Liver Cancer stage C (BCLC-C), with 70.4% having MVI and 46.6% presenting with extrahepatic metastasis. Previous treatments mainly included transcatheter arterial chemoembolization (74.7%), surgical resection (34.7%), and systemic therapy (24.0%). For those patients on systemic therapy, 2 received lenvatinib, 2 received tyrosine kinase inhibitor plus antibody against programmed cell death 1 (anti-PD1), 3 received sorafinib, and 5 received anti-PD1 alone.

### Follow-up outcomes

**Fig. 2:**
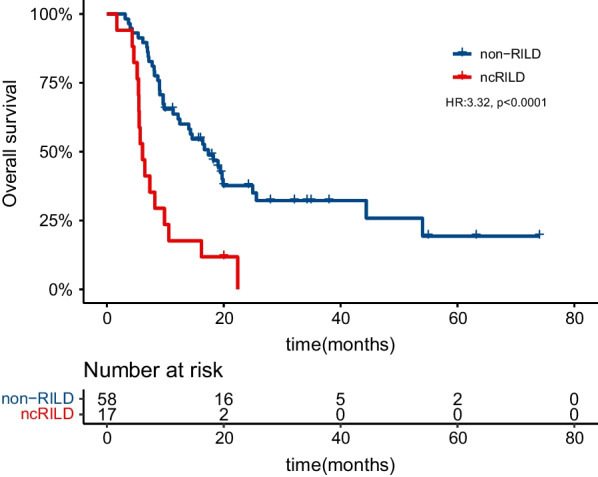
Survival curves of patients with RILD. RILD, radiation-induced liver disease

The median follow-up after completion of IMRT was 15.4 months for all patients, 7.9 months for the 17 patients with ncRILD, and 17.6 months for the 58 patients without ncRILD. The median OS was 9.0 and 24.2 months for patients with and without ncRILD, respectively (HR 3.32; 95% CI 1.81–6.08; *p* < 0.0001; Fig. 2), and 12.2 months for all patients. Cox univariate analysis revealed that surgical resection before IMRT, and ncRILD were prognostic factors, while other variables had no significant effect on prognosis (Table 2). Multivariate analysis further showed that only ncRILD is independent risk factors for OS (Table 2). In addition, the median OS was 10.0 and 17.0 months for patients with and without MVI, respectively (HR 1.65; 95% CI 0.90–3.00; *p* = 0.104); the median OS was 12.2 and 11.6 months for patients with CP-B7 and CP-B score ≥ 8, respectively (HR 1.22; 95% CI 0.65–2.28; *p* = 0.54). Of the 17 patients with ncRILD, 2 died of ncRILD, and 15 died of other causes.

**Table 2:** Univariate and multivariate Cox regression analyses associated with the risk of overall survival

Variable	Univariate analysis			Multivariate analysis		
	HR	95%CI	*p* value	HR	95%CI	*p* value
Sex, male versus female	1.22	0.44–3.40	0.701			
Age (years)	0.98	0.95–1.01	0.242			
Bodyweight (kg)	1.00	0.98–1.03	0.883			
Radiographic liver cirrhosis	1.37	0.80–2.34	0.252			
*Hepatitis etiology*						
Hepatitis B virus	0.70	0.33–1.49	0.358			
Hepatitis C virus	1.81	0.56–5.84	0.322			
Other	1.16	0.49–2.72	0.733			
*ECOG PS*	0.87	0.46–1.65	0.667			
0						
1						
2						
Total bilirubin (μmol/L)	1.00	0.99–1.01	0.935			
Albumin (g/L)	0.98	0.90–1.06	0.575			
PT (sec)	1.00	0.84–1.17	0.954			
*Child–Pugh score*	1.10	0.67–1.79	0.707			
7						
8						
9						
ALBI score	1.20	0.56–2.58	0.641			
ALBI Grade, 2 versus 3	1.46	0.76–2.79	0.252			
AST (U/L)	1.00	1.00–1.00	0.623			
ALT (U/L)	1.00	1.00–1.00	0.923			
ALP (U/L)	1.00	1.00–1.00	0.441			
WBC (10^12/L)	1.00	0.92–1.08	0.986			
HGB (g /L)	1.00	0.99–1.01	0.769			
PLT (10^9/L)	1.00	1.00–1.00	0.238			
ANC (10^9/L)	0.97	0.92–1.03	0.360			
ALC (10^9/L)	0.86	0.50–1.49	0.588			
Alpha fetoprotein (ng/ml), < 400 versus ≥ 400	1.57	0.92–2.69	0.101			
Max tumor size (cm)	1.03	0.99–1.09	0.170			
Tumor number, ≥ 3 versus < 3	1.49	0.87–2.55	0.148			
Macrovascular invasion, yes versus no	1.65	0.90–3.00	0.104			
Extrahepatic metastasis, yes versus no	0.98	0.57–1.68	0.936			
*BCLC stage*	1.28	0.79–2.07	0.312			
A						
B						
C						
Dose per fraction	0.98	0.72–1.33	0.875			
GTV (cc/ml)	1.00	1.00–1.00	0.372			
EQD2^8^ (Gy)	1.00	0.98–1.03	0.653			
NLV (cc/ml)	1.00	1.00–1.00	0.573			
Dmean (Gy)	1.04	0.99–1.09	0.143			
*Prior treatment*						
TACE	1.71	0.88–3.32	0.116			
RFA	0.33	0.08–1.37	0.128			
Surgical resection	0.54	0.30–0.97	0.039	0.60	0.33–1.08	0.088
Systemic therapy	0.65	0.34–1.21	0.211			
ncRILD	3.32	1.81–6.08	< 0.0001	3.08	1.67–5.66	< 0.001

ALBI, albumin–bilirubin; ALC, absolute lymphocyte count; ALP, alkaline phosphatase; ALT, alanine aminotransferase; ANC, absolute neutrophil count; AST, aspartate aminotransferase; BCLC, Barcelona Clinic Liver Cancer; CI, confidence interval; Dmean, mean dose to the normal liver; ECOG PS, Eastern Cooperative Oncology Group-performance status; EQD2, equivalent dose in 2‑Gy fractions; GTV, gross tumor volume; HGB, hemoglobin; NLV, normal liver volume; OR, odds ratio; PLT, platelets; PT, prothrombin time; RFA, radiofrequency ablation; RT, radiotherapy; TACE, transcatheter chemoembolization; WBC, white blood cells; ^8^, using LQ model, α/β = 8 Gy

### Incidence of ncRILD and treatment-related hepatotoxicity

Four patients with ncRILD were excluded; two because of tumour progression, and two because of viral reactivation (Fig. 1). Ultimately, the evolution of RILD was evaluated in 75 patients. Within three months of RT completion, the incidence of ncRILD was 22.7% (17/75). None of the patients were diagnosed with cRILD. The CP score increased in 28 patients (37.3%); 15 patients (20.0%) exhibited an increase of two or more. Eight patients (10.4%) shifted from CP-B to CP-C. No grade 4/5 laboratory adverse events of liver function were observed. The post-IMRT hepatotoxicity metrics and ncRILD incidence are shown in Table 3.

**Table 3:** Post-RT hepatotoxicity metrics

Variable	n	%
Liver function metrics		
CP Score + 1 or more	28	37.3
CP Score + 2 or more	15	20.0
CP Class Change	8	10.4
ALBI Grade + 1 or more	11	14.6
CTCAE 5.0 laboratory toxicities		
T-Bili ≥ G2	10	13.3
T-Bili G3	4	5.3
AST ≥ G2	7	9.3
AST G3	3	4.0
ALT ≥ G2	5	6.7
ALT G3	2	2.7
ALP ≥ G2	3	4.0
ALP G3	0	0
PLT ≥ G2	17	22.7
PLT G3	7	9.3
ncRILD	17	22.7

Data are n (%)

ALP, alkaline phosphatase; ALT, alanine aminotransferase; AST, aspartate aminotransferase; CP, Child–Pugh; CTCAE 5.0, the Common Terminology Criteria for Adverse Events of the National Cancer Institute v5.0; PLT, platelets; G1, grade1; G2, grade2; G3, grade3; ncRILD, non-classic radiation-induced liver disease; RT, radiotherapy; T-Bili, total bilirubin

### Variables associated with ncRILD risk

The cut-off points for GTV volume, EQD2^8^, NLV, and D_mean_ were 257.9 mL, 54.3 Gy, 565.2 mL, and 15.1 Gy, respectively, determined using Youden’s index and ROC curve analysis. Several clinical factors, including pre-treatment PT (pre-PT, ≤ 13 s vs. > 13 s, *p* = 0.016), tumour number (< 3 vs. ≥ 3, *p* = 0.029), and D_mean_ (< 15.1 Gy vs. ≥ 15.1 Gy, *p* = 0.028), correlated with ncRILD incidence in the univariate analysis. The multivariate analysis indicated that pre-PT (*p* = 0.035), tumour number (*p* = 0.036), and D_mean_ (*p* = 0.031) were also independent risk factors of ncRILD (Table 4). The cut-off value for D_mean_ (15.1 Gy) was an appropriate tolerance dose. The 75 patients with CP-B HCC were divided into two subgroups: those with D_mean_ < 15.1 Gy (27 cases) and those with D_mean_ ≥ 15.1 Gy (48 cases); the incidence of ncRILD in each group was 7.4% (2/27) and 31.3% (15/48), respectively.

**Table 4:** Univariate and multivariate analysis of parameters associated with the risk of ncRILD

Variable	Univariate analysis			Multivariate analysis		
	OR	95%CI	*p* value	OR	95%CI	*p* value
Sex, male versus female	1.51	0.16–13.88	0.716			
Age (years)	1.02	0.96–1.08	0.558			
Bodyweight (kg)	1.03	0.98–1.08	0.216			
Radiographic liver cirrhosis	0.83	0.28–2.45	0.735			
Hepatitis etiology						
Hepatitis B virus	1.03	0.19–5.49	0.973			
Hepatitis C virus	0	0.00-Inf	0.991			
Other	1.41	0.25–8.03	0.696			
*ECOG PS*						
0	Reference	–	–			
1	1.06	0.30–3.78	0.931			
2	0	0.00-Inf	0.992			
Total bilirubin (μmol/L), > 21 versus ≤ 21	1.38	0.47–4.09	0.556			
Albumin (g/L), ≥ 35 versus < 35	4,751,306	0.00-Inf	0.991			
PT (sec), > 13 versus ≦ 13	4.60	1.34–15.85	0.016	4.15	1.11–15.53	0.035
*Child–Pugh score*						
7	Reference	–	–			
8	0.58	0.12–2.96	0.516			
9	1.61	0.14–19.08	0.707			
ALBI score	1.16	0.25–5.38	0.849			
ALBI Grade, 2 versus 3	1.78	0.52–6.11	0.359			
AST (U/L), > 40 versus ≤ 40	1.00	0.30–3.26	0.994			
ALT (U/L), > 40 versus ≤ 40	0.91	0.31–2.70	0.871			
ALP (U/L), > 150 versus ≤ 150	2.95	0.92–9.47	0.068			
WBC (10^12/L), ≥ 4 versus < 4	1.48	0.4–5.48	0.56			
HGB (g /L), ≥ 110 versus < 110	1.69	0.55–5.17	0.359			
PLT (10^9/L), ≥ 100 versus < 100	1.17	0.28–4.90	0.833			
ANC (10^9/L), ≥ 2 versus < 2	2.89	0.58–14.45	0.195			
ALC (10^9/L), ≥ 0.8 versus < 0.8	0.68	0.2–2.39	0.551			
Alpha fetoprotein (ng/ml), < 400 versus ≥ 400	1.21	0.41–3.56	0.735			
Max tumor size (cm)	1.02	0.92–1.14	0.686			
Tumor number, ≥ 3 versus < 3	5.68	1.19–27.15	0.029	5.84	1.12–30.29	0.036
Macrovascular invasion, yes versus no	2.27	0.58–8.88	0.237			
Extrahepatic metastasis, yes versus no	1.89	0.63–5.65	0.257			
*BCLC stage*						
A	Reference	–	–			
B	42,544,812	0.00-Inf	0.991			
C	13,347,392	0.00-Inf	0.992			
Dose per fraction, Gy, ≥ 2.7 versus < 2.7	1.95	0.61–6.25	0.261			
GTV (cc/ml), ≥ 257.9 versus < 257.9	1.58	0.49–5.07	0.445			
EQD2^8^ (Gy), ≥ 54.3 versus < 54.3	1.29	0.42–3.98	0.653			
NLV (cc/ml), ≥ 565.2 versus < 565.2	4,837,693	0.00-Inf	0.991			
Dmean (Gy), ≥ 15.1 versus < 15.1	4.00	1.16–13.74	0.028	4.31	1.14–16.28	0.031
*Prior treatment*						
TACE	1.13	0.32–4.02	0.846			
RFA	0.66	0.07–6.09	0.716			
Surgical resection	0.50	0.15–1.74	0.278			
Systemic therapy	0.97	0.27–3.45	0.959			

ALBI, albumin–bilirubin; ALC, absolute lymphocyte count; ALP, alkaline phosphatase; ALT, alanine aminotransferase; ANC, absolute neutrophil count; AST, aspartate aminotransferase; BCLC, Barcelona Clinic Liver Cancer; CI, confidence interval; Dmean, mean dose to the normal liver; ECOG PS, Eastern Cooperative Oncology Group-performance status; EQD2, equivalent dose in 2‑Gy fractions; GTV, gross tumor volume; HGB, hemoglobin; NLV, normal liver volume; OR, odds ratio; PLT, platelets; PT, prothrombin time; RFA, radiofrequency ablation; RT, radiotherapy; TACE, transcatheter chemoembolization; WBC, white blood cells; ^8^, using LQ model, α/β = 8 Gy

### Dosimetric variables associated with ncRILD risk

Vx was defined as the percentage of normal liver volume receiving *x* Gy or more, and Vsx, as the liver volume (mL) receiving less than *x* Gy [19]. Dosimetric characteristics (V5, V7.5, V10, V15, V20, V25, V30, V35, Vs5, Vs7.5, Vs10, Vs15, Vs20, Vs25, Vs30, and Vs35) had cut-off values for ncRILD of 62.0%, 53.8%, 44.4%, 33.5%, 26.8%, 22.8%, 19.1%,15.8%, 60.7 mL, 399.3 mL, 484.1 mL, 603.2 mL, 668.3 mL, 760.9 mL, 816.8 mL, and 927.0 mL, respectively, as determined by ROC curve analysis. Seven variables, V5 (*p* = 0.039), V7.5 (*p* = 0.016), V10 (*p* = 0.016), V15 (*p* = 0.042), V25 (*p* = 0.042), V30 (*p* = 0.023), and V35 (*p* = 0.023) were significantly associated with ncRILD, according to univariate logistic regression analysis. However, multivariate analysis indicated that V5, V7.5, V10, V15, V25, V30, and V35 did not significantly predict ncRILD (Additional file 1: Table).

### Variables predicting ncRILD probability

**Fig. 3:**
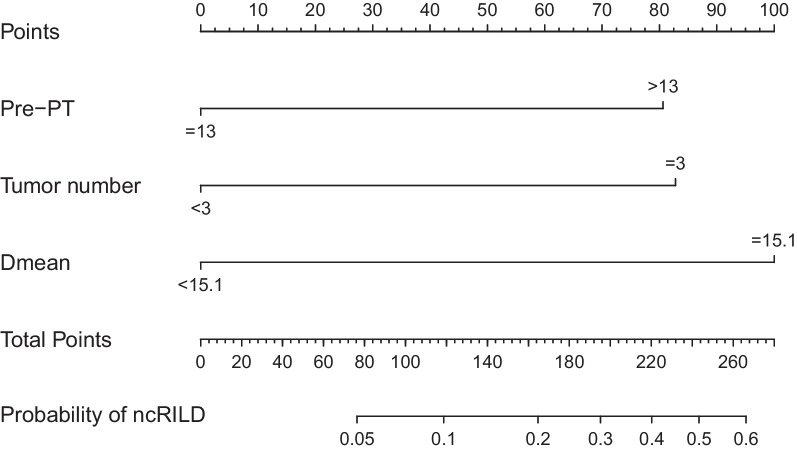
Nomogram for predicting the probability of ncRILD. The total number of points for each patient is used to predict the probability of ncRILD. Dmean, mean dose to the normal liver; ncRILD, non-classic radiation-induced liver disease; Pre-PT, pre-treatment prothrombin time

**Fig. 4:**
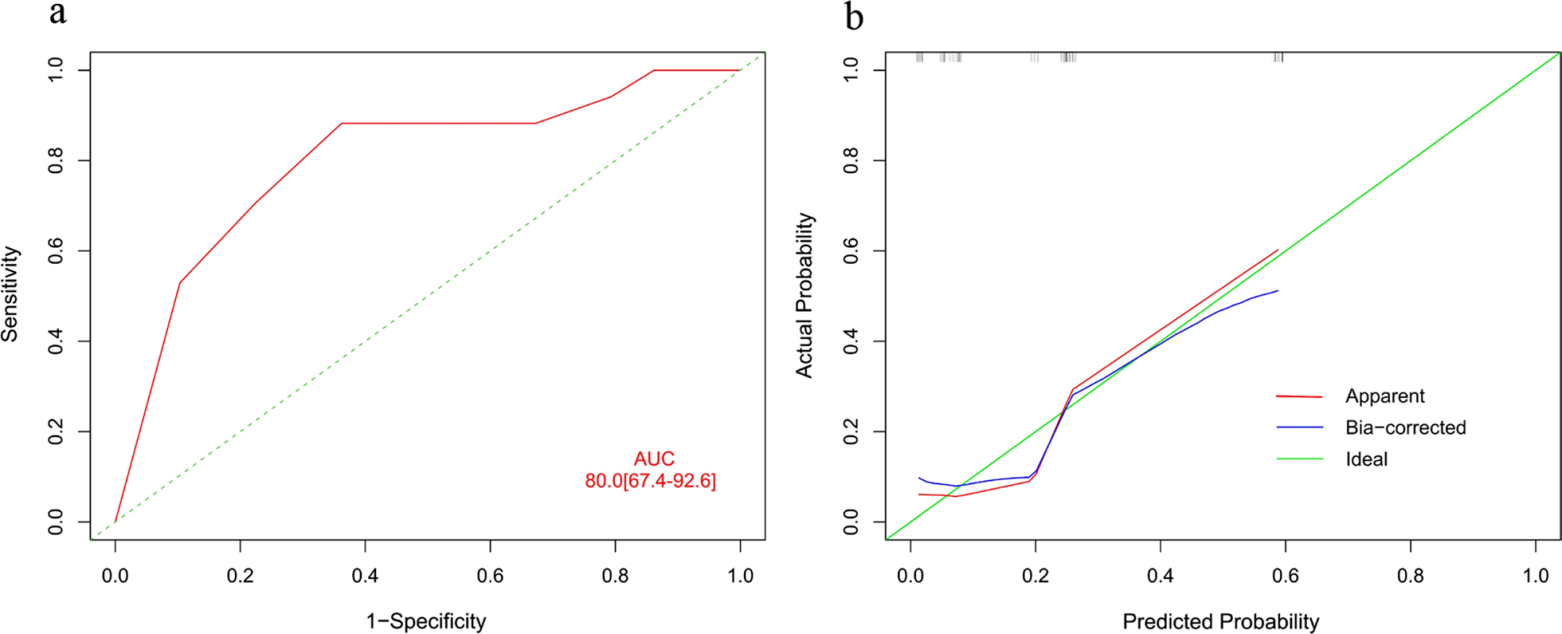
ROC curves and calibration curves indicating the ability of the nomogram to predict ncRILD. **a** ROC curves and **b** calibration curves. AUC, area under the receiver operating characteristic curve; ncRILD, non-classic radiation-induced liver disease; ROC, receiver operating characteristic

The prognostic nomogram integrated three significant prognostic factors, identified via multivariate analysis, for predicting the probability of ncRILD (Fig. 3). The nomogram model had a good predictive ability (AUC = 0.800; 95% CI 0.674–0.926; Fig. 4a), and the calibration plot for the risk of ncRILD showed an optimal agreement between prediction and observation (Fig. 4b).

## Discussion

RT has been shown to be safe and effective among patients with CP-A HCC [20, 21]; more cases of RILD, which was considered a major limitation in the application of radiation for HCC [9], occur among patients with CP-B HCC [20]. RILD was mostly reported in patients with CP-B HCC after SBRT or conformal radiotherapy (CRT) [10, 13, 22]; few studies have examined the incidence of RILD following 3-dimensional conformal radiotherapy (3DCRT) or IMRT [12]. However, a prediction model for RILD after IMRT in CP-B patients with locally advanced HCC has not been established. Therefore, we analyzed the incidence of RILD after IMRT in CP-B patients with locally advanced HCC, and established a model to predict the probability of RILD in these patients. We defined RILD according to the ncRILD criteria, because cRILD did not occur in our study.

A meta-analysis showed the median OS of advanced HCC patients with CP-B that received sorafenib as first-line therapy was 4.6 months [23], and the GIDEON study found similar poor survival [24]. For regorafenib as second-line treatment, the media OS of CP-B HCC patients was 4.6 months, which was equally disappointing [25]. Culleton et al. found that a median OS after SBRT for HCC patients was 9.9 months in CP-B7 and 2.8 months in CP-B score ≥ 8 [13]. They suggested SBRT is a treatment option for HCC patients with modestly impaired (CP-B7) liver function. In addition, one study found that the median survival of 184 HCC patients with CP-B treated with fractionated conformal RT was 9.4 months, 10.7 months in CP-B7, 9.1 months in CP-B8, and 5.6 months in CP-B9 [12]. We found that IMRT led to better outcomes with the median OS of 12.2 months for all patients in this study, 12.2 months for CP-B7 patients, and 11.6 months for CP-B score ≥ 8, suggesting that IMRT can benefit the CP-B patients with locally advanced HCC. Of note, MVI was present in 53 of 75 (70.7%) patients in our series, meaning that they were more prone to develop progressive disease and had poorer prognoses [26]. Similarly, Fang et al. showed a considerable survival in 3D-CRT (1-, 2-, and 3-year OS: 54.0%, 33.0% and 18.0%) in 134 HCC patients with portal vein tumor thrombus (PVTT). They suggested CP-B is independent predictor of poor prognosis in HCC with PVTT [27].

RILD is a major form of dose-limiting toxicity that can ultimately cause liver failure and lead to high risk in death [28]. Sun et al. reported that ncRILD was a most significant factor for OS after fractionated conformal RT for HCC patients [12], which are consistent in our series. In this study, the median OS of patients without ncRILD was significantly better than that of patients with ncRILD, and ncRILD is the only independent risk factor for OS in CP-B HCC patients treated with IMRT based on multivariate Cox regression analysis. Therefore, ncRILD may has a significant effect on the survival of CP-B patients with locally advanced HCC.

The results of prior studies on the incidence of RILD in patients with CP-B HCC are inconsistent [12, 20, 29, 30]. In one study on the incidence of RILD after SBRT (40–60 Gy in 3–5 fractions), RILD was observed in 19 of the 28 patients (67.9%) with CP-B HCC [29]. A hypofractionated 3DCRT study reported an incidence of cRILD or ncRILD of 56% (9/16) in patients with CP-B HCC receiving 40–60 Gy, with a fraction size of 4–8 Gy [20]. These studies suggest that patients with CP-B HCC do not tolerate RT. In contrast, another study reported that the incidence of ncRILD was 19.7% among 132 evaluable patients with CP-B HCC after fractionated CRT [12]. In our study, the incidence of ncRILD was 22.7% (17/75) after IMRT, with a median fraction size of 3 Gy, indicating that the incidence of RILD in CP-B patients with locally advanced HCC following IMRT is acceptable.

Many researchers have reported various clinical and dosimetric parameters that predict RILD [21, 29, 31]. A study of SBRT for patients with CP-A HCC reported that higher CP scores and liver doses, including the mean dose, were associated with liver toxicity that resulted in an increase in the CP score of ≥ 2 points [22]. Another study of SBRT showed that CP score was an important factor in determining RILD risk [29]. A hypofractionated three-dimensional conformal RT study reported that V20 was a predictor of RILD in patients with CP-A cirrhosis [21]. In addition, RT technique and some dosimetric parameters are associated with the risk of ncRILD after fractionated conformal RT [12]. NLV is the most predictive dosimetric parameter of ncRILD in patients with CP-B, according to a multivariate analysis. However, no independent risk factors of ncRILD have been identified, and the development of a stable model for predicting ncRILD in patients with CP-B HCC is urgently needed [9]. In the present study, pre-PT, tumour number, and D_mean_ were significantly associated with ncRILD incidence in the multivariate analysis. Moreover, we built a novel predictive modeling system using nomogram methodology to predict ncRILD probability in CP-B patients with locally advanced HCC treated with IMRT. The ncRILD predictions of the nomogram were supported by an AUC of 0.800 and calibration curves, suggesting that the nomogram can well predict ncRILD. The ability of the nomogram to reliably predict outcomes before RT enables the selection of optimal treatment options and accurate assessments of prognosis.

This study had several limitations. First, most HCC cases in this study were due to HBV infection, which may correlate with a worse prognosis [32]. However, to minimize the effect of this limitation, we only analyzed cases without viral replication or tumour progression after IMRT. Second, the study had a relatively small sample size and lacked an available independent validation study population. Our nomogram needs to be validated in larger populations in the future. Third, this is a retrospective study. Fourth, the patients were not treated with anti-coagrant agents. In addition, our study is possible missed information in the ineligible patients. Further well-designed prospective studies should be conducted to overcome these research gaps.

## Conclusions

CP-B patients with locally advanced HCC treated with IMRT exhibited favorable OS and an acceptable ncRILD incidence. The nomogram including D_mean_, pre-PT, and tumour number accurately predicted ncRILD probability and can help clinical doctors cautiously applied individualized treatment approach after IMRT in CP-B patients with locally advanced HCC.

## Supplementary Information

**Additional file 1. Table**: Univariate and multivariate analysis of dosimetric parameters associated with the risk of ncRILD.

## Data Availability

The data underlying this article will be shared on reasonable request to the corresponding author.

## Abbreviations

AFP: Alpha-fetoprotein
ALB: Albumin
ALBI: Albumin-bilirubin
ALP: Alkaline phosphatase
ALT: Alanine aminotransferase
AST: Aspartate aminotransferase
CP-B: Child–Pugh grade B
cRILD: Classic radiation-induced liver disease
CRT: Conformal radiotherapy
CT: Computed tomography
CTCAE 5.0: Common Terminology Criteria for Adverse Events of the National Cancer Institute v5.0
GTV: Gross tumour volume
HBV: Chronic hepatitis B virus infection
HCC: Hepatocellular carcinoma
IMRT: Intensity-modulated radiotherapy
ncRILD: Non-classic radiation-induced liver disease
OS: Overall survival
PT: Prothrombin time
RILD: Radiation-induced liver disease
RT: Radiotherapy
TACE: Transcatheter arterial chemoembolization
TB: Total bilirubin
