# Supplementary material for: Non-classic radiation-induced liver disease after intensity-modulated radiotherapy for Child–Pugh grade B patients with locally advanced hepatocellular carcinoma
Source: Radiat Oncol. 2023 Mar 8;18:48. doi: 10.1186/s13014-023-02232-5 (PMC9993633; doi:10.1186/s13014-023-02232-5)
Supplement: Supplementary file 1 — Additional file 1. Table: Univariate and multivariate analysis of dosimetric parameters associated with the risk of ncRILD. [file 13014_2023_2232_MOESM1_ESM.docx]

Supplemental Table. Univariate and multivariate analysis of dosimetric parameters associated with the risk of ncRILD.

| Variable | Univariate analysis | | | Multivariate analysis | | |
| --- | --- | --- | --- | --- | --- | --- |
|  | OR | 95%CI | *p* value | OR | 95%CI | *p* value |
| V5(%), ≥62.0 vs＜62.0 | 9.08 | 1.12-73.41 | 0.039 | 2.08 | 0.06-75.36 | 0.690 |
| V7.5(%), ≥53.8 vs＜53.8 | 13.00 | 1.62-104.64 | 0.016 | 2.70 | 0.01-968.96 | 0.741 |
| V10(%), ≥44.4 vs＜44.4 | 13.00 | 1.62-104.64 | 0.016 | 7.66 | 0.03-1785.60 | 0.464 |
| V15(%), ≥33.5 vs＜33.5 | 4.06 | 1.05-15.67 | 0.042 | 0.09 | 0.00-2.67 | 0.162 |
| V20(%), ≥26.8 vs＜26.8 | 2.64 | 0.77-9.07 | 0.123 |  |  |  |
| V25(%), ≥22.8 vs＜22.8 | 4.06 | 1.05-15.67 | 0.042 | 2.18 | 0.06-80.17 | 0.671 |
| V30(%), ≥19.1vs＜19.1 | 6.09 | 1.28-29.10 | 0.023 | 3.86 | 0.11-138.53 | 0.460 |
| V35(%), ≥15.8 vs＜15.8 | 6.09 | 1.28-29.10 | 0.023 | 3.86 | 0.11-138.53 | 0.460 |
| Vs5(mL), ≥60.7 vs＜60.7 | 2.56 | 0.30-22.06 | 0.392 |  |  |  |
| Vs7.5(mL), ≥399.3 vs＜399.3 | 1.33 | 0.44-4.02 | 0.614 |  |  |  |
| Vs10(mL), ≥484.1 vs＜484.1 | 1.44 | 0.47-4.36 | 0.522 |  |  |  |
| Vs15(mL), ≥603.2 vs＜603.2 | 1.44 | 0.47-4.36 | 0.522 |  |  |  |
| Vs20(mL), ≥668.3 vs＜668.3 | 1.44 | 0.47-4.36 | 0.522 |  |  |  |
| Vs25(mL), ≥760.9 vs＜760.9 | 1.43 | 0.45-4.52 | 0.54 |  |  |  |
| Vs30(mL), ≥816.8vs＜816.8 | 1.43 | 0.45-4.52 | 0.54 |  |  |  |
| Vs35(mL), ≥927.0 vs＜927.0 | 3.26 | 0.77-13.88 | 0.11 |  |  |  |

OR, odds ratio; CI, confidence interval; ncRILD, non-classic radiation-induced liver disease.

Vx, the percentage of normal liver volume receiving > x Gy radiation; Vsx, the absolute liver volume (mL) spared from < x Gy radiation.
